# Assessment of Exposure to VOCs among Pregnant Women in the National Children’s Study

**DOI:** 10.3390/ijerph13040376

**Published:** 2016-03-29

**Authors:** Elizabeth Barksdale Boyle, Susan M. Viet, David J. Wright, Lori S. Merrill, K. Udeni Alwis, Benjamin C. Blount, Mary E. Mortensen, John Moye, Michael Dellarco

**Affiliations:** 1 Health Studies, Westat, 1600 Research Blvd, Rockville, MD 20850, USA; SusanViet@westat.com (S.M.V.); DavidWright@Westat.com (D.J.W.); LoriMerrill@westat.com (L.S.M.); 2 Tobacco and Volatiles Branch, Division of Laboratory Sciences, Centers for Disease Control and Prevention, Atlanta, GA 30341, USA; eoe3@cdc.gov (K.U.A.); bkb3@cdc.gov (B.C.B.); 3 Division of Laboratory Sciences, National Center for Environmental Health, Centers for Disease Control and Prevention, Atlanta, GA 30341, USA; zeo4@cdc.gov; 4 *Eunice Kennedy Shriver* National Institute of Child Health and Human Development, Bethesda, MD 20892, USA; moyej@exchange.nih.gov (J.M.Jr.); dellarcom@mail.nih.gov (M.D.)

**Keywords:** birth cohort study, pregnant women, tobacco, volatile organic compounds, urinary metabolites, National Children’s Study

## Abstract

Epidemiologic studies can measure exposure to volatile organic compounds (VOCs) using environmental samples, biomarkers, questionnaires, or observations. These different exposure assessment approaches each have advantages and disadvantages; thus, evaluating relationships is an important consideration. In the National Children’s Vanguard Study from 2009 to 2010, participants completed questionnaires and data collectors observed VOC exposure sources and collected urine samples from 488 third trimester pregnant women at in-person study visits. From urine, we simultaneously quantified 28 VOC metabolites of exposure to acrolein, acrylamide, acrylonitrile, benzene, 1-bromopropane, 1,3-butadiene, carbon disulfide, crotonaldehyde, cyanide, *N,N*-dimethylformamide, ethylbenzene, ethylene oxide, propylene oxide, styrene, tetrachloroethylene, toluene, trichloroethylene, vinyl chloride, and xylene exposures using ultra high performance liquid chromatography coupled with an electrospray ionization tandem mass spectrometry (UPLC-ESI/MSMS) method. Urinary thiocyanate was measured using an ion chromatography coupled with an electrospray ionization tandem mass spectrometry method (IC-ESI/MSMS). We modeled the relationship between urinary VOC metabolite concentrations and sources of VOC exposure. Sources of exposure were assessed by participant report via questionnaire (use of air fresheners, aerosols, paint or varnish, organic solvents, and passive/active smoking) and by observations by a trained data collector (presence of scented products in homes). We found several significant (*p* < 0.01) relationships between the urinary metabolites of VOCs and sources of VOC exposure. Smoking was positively associated with metabolites of the tobacco constituents acrolein, acrylamide, acrylonitrile, 1,3-butadiene, crotonaldehyde, cyanide, ethylene oxide, *N,N*-dimethylformamide, propylene oxide, styrene, and xylene. Study location was negatively associated with the toluene metabolite *N*-acetyl-*S*-(benzyl)-l-cysteine (BMA), and paint use was positively associated with the xylene metabolites 2-methylhippuric acid (2MHA) and 3-Methylhippuric acid & 4-methylhippuric acid (3MHA + 4MHA). A near-significant (*p* = 0.06) relationship was observed between acrylamide metabolites and observation of incense.

## 1. Introduction

Volatile organic compounds (VOCs) are compounds with low boiling points; VOC exposures originate from numerous natural and anthropogenic sources such as cleaning products, paints, solvents, personal care products, automotive exhaust, and tobacco smoke [1,2,3]. Exposure to certain VOCs may increase the risk for birth defects, neurocognitive impairment, asthma, and cancer [4,5,6,7]. Due to these health concerns and ubiquitous exposure, the impact of exposure to VOCs on child health and development is often studied [8,9,10]. Table 1 lists some common exposure sources for selected VOCs.

Epidemiologic studies designed to evaluate these research questions can use a variety of exposure assessment methods, such as questions about use of products or behaviors that may increase exposure to certain VOCs, measurement of VOCs in blood, measurement of metabolites in urine, and measurement of VOCs in indoor air. Each of these exposure assessment methods has advantages and disadvantages. Questionnaires are inexpensive to administer, but when used as the sole source of exposure assessment, their accuracy is dependent on participant recall and knowledge of participant behavior [14,15]. Observations of VOC sources in homes by trained data collectors are not subject to recall bias, but are limited in that some household characteristics may be modified by the participant prior to the study home visit. Blood VOCs provide a scientifically sound basis to extract a dose response, but have very short biological half-lives [3,16]. Metabolites of VOCs in urine have a longer biological half-life than VOCs in blood and are more stable during storage and handling, but most metabolites still have half-lives of less than two days and thus some intermittent exposures may be missed [3]. Assessment of exposure to VOCs by sampling air has been completed in many studies, but pharmacologic and physiologic parameters need to be accounted for in sophisticated models to estimate internal absorbed dose [16].

The U.S. National Children’s Study (NCS), a planned longitudinal cohort study that was to recruit participants from across the United States from before birth through age 21, measured exposure to VOCs [17,18]. During the initial phase of the Vanguard Study from 2009 to 2010, a variety of methods both indirect and direct were included to assess exposure to VOCs: Indirect methods, questionnaires and observations of VOC sources in the home. Direct methods refer to, measurement of VOC metabolites in urine, and measurement of VOCs in air and water in a subsample of homes [18]. In this paper we compare some of the indirect exposure assessments used in the NCS with the measurement of metabolites of VOCs in urine. Several other studies have compared indirect methods of estimating VOC exposure with VOC measurements in blood or air [1,19,20,21,22], but this is the first study to compare these methods with a large panel of urinary metabolites of VOC exposure. 

## 2. Methods

### 2.1. Study Population

As described elsewhere [18,23,24], 1399 women were enrolled in the NCS initial Vanguard Study from 2009 to 2010 from seven locations: Queens County, New York; Duplin County, North Carolina; Salt Lake County, Utah; Orange County, California; Montgomery County, Pennsylvania; Waukesha County, Wisconsin; and four adjacent counties in South Dakota and Minnesota (Brookings County, SD; Yellow Medicine County, MN; Pipestone County, MN; Lincoln County, MN) [14]. Enrolled women could have completed at least one of several different types of study visits during the NCS initial Vanguard Study: pre-pregnancy visit (P1), first trimester visit (T1), third trimester visit among women who had a prior study visit (T3-Prior), and third trimester visit among women who did not have a prior study visit (T3-First). All home visits included environmental sample collections such as air, water and dust [18], an interview, collection of biospecimens, and a physical exam. P1, T1, and T3-First visits also included an observational walk-through of the residence. Participants could refuse any portion of a study visit. There were a total of 488 unique participants with interviews, observations, and urine VOCs results: 344 had T3-Prior visits, and 144 had T3-First visits.

All biospecimens were collected in accordance with pre-tested standard operating procedures which were the same at all study locations [25]. A portion of biospecimens collected during the initial Vanguard Study was analyzed through a collaborative pilot study between the NCS and the Centers for Disease Control and Prevention (CDC) [26,27]. In this collaborative pilot, biomarkers of exposure to environmental chemicals were measured (Environmental Health Laboratory, CDC) in biospecimens collected from a sample of pregnant Vanguard Study enrollees. Most specimens used in the pilot were obtained from the third trimester visit (T3-Prior or T3-First) and consisted of a sample of about 70 women from each study location [27].

Written informed consent was obtained from all participants and the study protocol was approved by the NICHD Institutional Review Board (IRB) and the IRBs at each Vanguard Study institution (NICHD IRB protocol number 09-CH-N083). The involvement of the CDC laboratory was determined not to constitute engagement in human subjects research, and thus was not reviewed by the CDC IRB.

### 2.2. Urine Collection and Analysis

All urine analytes were measured in aliquots of spot urine samples that were collected at study visits into pre-screened, metal-free containers. Urine samples were shipped frozen to the NCS Biological and Environmental Sample Repository (Fisher Bioservices; Rockville, MD, USA), where they were aliquoted and frozen at −150 °C. Samples were shipped to the CDC for analysis on dry ice and stored locally at −20 °C until they were analyzed. The urine aliquots were analyzed simultaneously for 28 urinary VOC metabolites using an ultra high performance liquid chromatography coupled with an electrospray ionization tandem mass spectrometry (UPLC-ESI/MSMS) method [3]. The limits of detection of this method range from 0.5 to 20 ng/mL. The 28 metabolites are biomarkers of exposure to acrolein, acrylamide, acrylonitrile, benzene, 1-bromopropane, 1,3-butadiene, carbon disulfide, crotonaldehyde, hydrogen cyanide, *N,N*-dimethylformamide, ethylbenzene, ethylene oxide, propylene oxide, vinyl chloride, styrene, tetrachloroethylene, toluene, trichloroethylene, and xylene. All of these parent compounds are tobacco smoke constituents, with the exception of 1-bromopropane, tetrachloroethylene, and trichloroethylene. The metabolite for cyanide measured by the urine VOC method, 2-aminothiazoline-4-carboxylic acid, is not specific for hydrogen cyanide exposure, thiocyanate (SCN), a more specific biomarker for hydrogen cyanide, was measured using a quantitative procedure for the measurement of nitrate, perchlorate, and thiocyanate in human urine using ion chromatography coupled with electrospray tandem mass spectrometry [28]. Urine creatinine was measured on a Roche/Hitachi Modular P Chemistry Analyzer, using a method similar to the one employed in the National Health and Nutrition Examination Survey [29].

### 2.3. Blood Collection and Analysis

Blood specimens were collected by trained phlebotomists in the home or clinic, into no-additive serum red top tubes (Becton Dickinson; Franklin Lakes, NJ, USA) for serum cotinine. Samples were centrifuged locally at ambient temperature within 2 h of collection and shipped on ice packs to the repository. There red top tubes were re-spun and aliquotted into pre-screened metal-free cryovials. Aliquots were stored at vapor phase liquid nitrogen temperature (−150 °C) until shipment on dry ice to the CDC. Serum cotinine was analyzed by the CDC’s tobacco exposure biomarkers laboratory using a standard method with a limit of detection of 0.015 ng/mL [30,31].

### 2.4. Questionnaire and Observation Data

The home observation form that was completed by a trained data collector at the P1, T1, and T3-First visits contained the following observations related to VOC exposure: air freshener; incense; candles; and other scented products (heavily scented soaps, *etc.*). Since the observations were not conducted at the T3-Prior visit, if the urine collected came from a T3-Prior visit, we used the observation data from the participant’s T1 visit.

At all visits, the interview contained a traditional data collector-led portion and an Audio Assisted Computer Self-Interview (ACASI). The data collector-led portion at the third trimester visits (T3-Prior and T3-First) asked participants about the use of products over the last three months that may contain or off-gas VOCs such as air fresheners, other aerosols, paint or varnish, paint thinners and other organic solvents. The ACASI interview asked about current smoking, the number of cigarettes per day, and hours around smoking. The T1 interview also included a question about the frequency of gas pumping. Thus, questionnaire items that were assessed at the same visit that the urine was collected include: current smoking and the reported use of VOC-containing products (*i.e.*, air fresheners, other aerosols, paint or varnish, paint thinners and other organic solvents).

Following all third trimester visits, the participants were asked to complete a three-day time, place and activity diary based on the diary used in the National Human Exposure Assessment Survey [32]. This diary was a self-administered, paper diary which was completed for three consecutive days, always including a weekend day and two weekdays. Each diary day was divided into half-hour intervals. The participant recorded her activity and location during each interval. From this diary data we created an average cooking time variable.

### 2.5. Statistical Methods

For this analysis, we modeled the relationship between the predictor variables and the concentration of the VOC metabolites, using a linear regression of log_10_ VOC (all predictor variables were used in each regression as main effects, *i.e.*, no interactions). We used maximum likelihood estimation (MLE) via the LIFEREG procedure in SAS *vs.* 9.1 (Cary, NC, USA) to account for left-censored data (*i.e.*, data below the limit of detection (LOD)) [33]. The urinary VOC metabolite data were highly right skewed (approximating a log-normal distribution), therefore a log_10_ transformation was applied. Concentrations of the six metabolites with very low percent detection (<25%) were not modeled, *i.e.*, *N*-acetyl-*S*-(1,2-dicholorovinyl)-l-cysteine (1DCVMA), *N*-acetyl-*S*-(2,2-dicholorovinyl)-l-cysteine (2DCVMA), *N*-acetyl*-S-*(trichlorovinyl)-l-cysteine (TCVMA), *N*-acetyl-*S*-(2,4-dimethylphenyl)-l-cysteine (DPMA), *N*-acetyl-*S*-(1-hydroxymethyl-2-propenyl)-l-cysteine (MHBMA1), *N*-acetyl*-S-*(2-hydroxy-3-butenyl)-l-cysteine (MHBMA2).

The 22 urinary log_10_ metabolite concentrations were used as dependent variables in regressions which included log_10_ creatinine as a predictor variable [34]. We chose which interview and observation variables to include in each model as predictor variables, based on knowledge of sources of exposure to the parent compounds and predictor variables remained in the final models regardless of significance. Model variables were handled in the following ways: study location was categorized as urban (Queens County, NY; Orange County, CA; Salt Lake City, UT; and Montgomery County, PA) *vs.* rural (South Dakota and Minnesota, Duplin County, NC; and Waukesha County, WI) based on the approach used in Boyle *et al.*, 2015 [18]. Observations of air fresheners, scented candles, and other scented products were categorized as present or not present. An indicator variable (*i.e.*, visit type) was used to indicate participants with first trimester visits or third trimester visits. Reported use of air fresheners, other aerosols, and paint/varnish, was categorized as every day, some days, or not at all/refused. Paint thinner and turpentine use were not included in the final models because use was rare (less than 1%) and all such users also used paint.

Smoking exposure was assigned based on both self-reported smoking status from the interview and serum cotinine. From the interview data, smokers and non-smokers were categorized into three mutually exclusive smoking categories: heavy smokers (10 or more cigarettes per day), moderate smokers (less than 10 cigarettes per day), and non-smokers. Self-reported smokers (moderate and heavy) and subjects with high serum cotinine levels (>3.0 ng/mL) were classified as smokers similar to the approach used with non-pregnant reference populations [35]. Non-smokers were classified as some second hand smoke exposure (>LOD (0.015 ng/mL)–3.0 ng/mL), or minimal second hand smoke exposure (<LOD). 

## 3. Results

### 3.1. Descriptive Statistics

Table 2 shows the VOC-related characteristics considered in regressions for the NCS 2009−2010 year among participants with non-missing urine VOCs samples (*n* = 488). The results were similar when the sample included participants with missing urine VOCs measurements. Among the four ”observed” variables, candles were most frequently observed (51.4%) while incense was least frequently observed in households (6.2%). Among the self-reported VOC categories, air fresheners were used by 72.3% of participants over the last three months, while 23.6% reported using paints or varnishes. Most participants reported cooking for less than one hour on average over the three time and place diary days. Self-reported smoking behavior indicated only 27 smokers among the 488 subjects. The population had low smoking and secondhand-smoke exposure; with only 25.8% having reported smoking, being exposed to smoke, or having a detectable cotinine result. Finally, the study population was close to evenly divided among rural and urban study locations. 

Observed air freshener and reported air freshener use were correlated. While the correlation was statistically significant and greater than zero (φ = 0.24), the correlation was not large. Self-reported air freshener use (72.3%) was much greater than observed air freshener presence (26.8%). Therefore, despite some potential confounding, both self-reported and observed air freshener were included in VOC metabolite regressions.

### 3.2. Metabolite Detection and Distribution

Table 3 presents the detection frequency, the median, the 75th percentile, and the maximum for the 22 VOC metabolites included in the regression models. Sixteen of the 22 metabolites had a detection frequency greater than 90%. Most VOC metabolites were highly right skewed with maximum observed levels generally 10-fold greater than the median.

### 3.3. Regression Results

Table 4 presents the regression results for all models. The most common significant covariate was smoking exposure. Smoking exposure predicted (*p* < 0.01) all VOC metabolites which are biomarkers of tobacco constituents except: PMA and MU (both benzene metabolites), TTCA (a carbon disulfide metabolite), PGA (an ethylbenzene and styrene metabolite), and BMA (a toluene metabolite). Smokers had increased VOC metabolite levels compared to subjects with none or minimal smoke exposure. VOC metabolite levels were similar among subjects with none or little smoke exposure and subjects with some smoke exposure (none of the 15 regressions showed statistically significant differences).

Paint or varnish use was considered a potential VOC metabolite covariate in five regressions (MA, PHEMA, BMA, 2MHA, and 3MHA + 4MHA). Paint or varnish use was associated with increased concentration of the xylene metabolites 2MHA and 3MHA + 4MHA. In both the 2MHA and 3MHA + 4MHA regressions participants who used paint products had increased metabolite levels compared to subjects that never used paint products (*p* < 0.0001 in both regressions).

Among other observed and reported product uses, no statistically significant results were found. Near-significant results (*p* = 0.06 in both regressions) were noted for observed incense use on two acrylamide metabolites (GAMA and AAMA). Subjects with incense observed in the household had increased GAMA and AAMA compared to subjects with no incense observed in the household.

The toluene metabolite BMA was associated with geographical location (*i.e.*, urban v. rural location). Concentrations of BMA among subjects living in rural PSUs were lower than those who lived in urban PSUs (*p* = 0.003). An indicator variable (*i.e.*, visit type) was included in all regressions to account for subjects where “observed” covariates were collected at the first trimester rather than the third trimester. 

Finally, in six regressions (DHBMA, MHBMA3, CEMA, 3HPMA, GAMA, and AAMA) we looked for a relationship between cooking and VOC exposure. In none of these regressions was cooking exposure statistically significant. Additional covariates considered in each regression but found to be not statistically significant are listed in Table 4.

## 4. Discussion

For the first time, data on the variability of the concentration of 22 urinary metabolites of 18 parent VOCs has been compared with observed and reported sources of exposure to VOCs in a geographically diverse sample of pregnant women in the United States. In this analysis we compared indirect exposure measurement methods such as questionnaires and observations of VOC sources in the home with a more direct exposure measurement method, the measurement of VOC metabolites in urine.

Recently, Jain reported VOC metabolite levels using data from the National Health and Nutrition Examination Survey (NHANES) 2011–2012 [2,36]. The metabolite detection rate and distribution presented here is similar to that found in NHANES 2011–2012 [2,36]. As observed in this study and NHANES, VOC exposure tends to be highly right skewed, and thus VOC exposure biomarkers such as urinary VOC metabolites are also highly right skewed. For this reason, we model our data using log_10_ transformed results to achieve more of a normal distribution. Additionally, we present the geometric mean as the central tendency for summary statistics.

Most of the metabolites were associated with smoking. These findings are not unexpected because the panel of measurements was designed to measure metabolites of VOCs that are present in tobacco smoke and thus the same increased exposures have been observed in NHANES [2,3,36]. We did not observe this relationship with smoking for the following metabolites: benzene metabolite PMA, the benzene metabolite MU, the carbon disulfide metabolite TTCA, the ethylbenzene and styrene metabolite PHGA, and the toluene metabolite BMA. However, BMA and MU are not specific biomarkers for tobacco constituents and both can be formed from exposure to benzyl alcohol, and benzyl acetate in personal care products, and sorbic acid (a food preservative) respectively [37]. Others have found a weak correlation between serum cotinine and TTCA as well as serum cotinine and PMA [3]. There are several logical reasons for these differences, TTCA is not specific to carbon disulfide exposure as it is also associated with the consumption of brassica vegetables [38]. PMA is specific to benzene exposure, but is only a minor benzene metabolite. The relatively low benzene exposures in this study may not have been of sufficient magnitude to be detected with our method [39,40].

We also observed a near-significant relationship between acrylamide metabolites and incense and a significant relationship between paint/varnish use and the xylene metabolites, supporting the use of indirect exposure measures such as questionnaires and observations for exposure to these chemicals. These associations are not unexpected because xylenes are common components of paints and paint thinners and acrylamide is a component of incense [41,42]. We did not observe statistically significant (*p* < 0.01) relationships between the observation of air fresheners, scented candles, other scented products, reported average cooking time, reported use of air fresheners and aerosols, and gas pumping to VOC metabolites in any models (Table 3). Possible explanations for this include: the products used did not contain VOCs or were made with VOCs we could not detect, the sporadic use of these products; the short biological half-lives of the VOC metabolites (2.1–34 h) [3]; the fact that many of the parent compounds having a wide variety of exposure sources; or observations and urine collection being completed at different visits in most cases.

We expected some relationships between VOC metabolites and the administered questions about used of scented products. Part of this expectation is based on findings from the Avon Longitudinal Study of Parents and Children (ALSPAC), which reported a relationship between the mothers’ report of the use of aerosols and scented products and the concentration of total VOCs in indoor air [43]. However, our analyses are quite different than these ALSPAC findings [43]. We looked at exposure to individual VOCs by measuring urinary metabolites whereas these ALSPAC findings present total VOCs in indoor air at multiple time points [43]. Urinary biomarkers represent an individual’s total exposure to the parent compounds of interest over a short time period, because the half-lives are roughly 2.1–34 h depending on the compound [3]. The air samples from the ALSPAC study were set up in the homes for 1 month, and up to 10 samples were received from each home. Since the use of air fresheners and aerosols may be a regular behavior, but not one that occurs daily, it makes sense that a relationship would be more likely to be found with an exposure measurement which represents a longer time period. Additionally, ALSPAC measured total VOCs using a non-specific method that would combine all volatiles into one single value, whereas we compared reported use of products with a selective measure of biomarkers of exposure to 20 targeted VOCs [43].

Exposure assessment using urinary metabolites has limitations, such as the metabolite being produced from exposure to multiple parent compounds or the metabolite being produced not from a parent-chemical uptake and metabolism but as a result of uptake of the metabolite from environmental media [44]. Additionally, within individual variability results from episodic and varied exposures, and thus, averaging exposure over a longer time frame would likely produce exposure distributions that are less highly skewed. Differences in metabolism between people may further contribute to variability, but our experience to date with these VOC metabolites leads us to believe that metabolism is unlikely to be as important as differences in exposure. Another limitation of our analysis is that most of the home observations and the question about gas pumping were not completed at the same point in visit that the urine samples were collected and these behaviors may have changed. Removing the samples where observations were not completed at the same visit would have severely lowered our statistical power, as the sample size would have been reduced from 488 to 144. Despite the time difference between observation completion and biospecimen collection for some study participants, we did observe one near-significant relationship between acrylamide exposure and the observation of incense. We also were unable to evaluate many possible factors which could have been related to some of the VOC sources of interest, such as the possibility of occupational exposures, time spent near road ways, or home proximity to gas stations. These factors were not evaluated in this study because a preliminary look at the results of the occupational questions revealed that manufacturing jobs were not common among women in this dataset and the home and work addresses of NCS participants was not available.

Concentrations of the toluene metabolite BMA were lower in rural locations than in urban locations, although the significance of this finding is uncertain given the lack of specificity of the BMA biomarker. BMA can be formed from multiple sources other than toluene (e.g., benzyl alcohol, a widespread constituent of cosmetic products including shampoo, creams, after-shave lotions, and fragrances) [45].

## 5. Conclusions

Exposure assessment in epidemiological studies is challenging and can be conducted via a variety of methods. In this analysis, we show that is feasible to assess for exposure to VOCs in pregnant women. We also observed some associations between indirect VOC exposure measures, use of paint/varnish, and observation of incense in the home and more direct exposure measure of VOC metabolites in urine. All of the exposure assessment methods discussed in this paper have unique strengths. Measurements of exposure in biospecimens can help us estimate internal dose, but only interview and observation data can inform studies of the likely sources of exposure to these chemicals.

## Figures and Tables

**Table 1:** Common exposure sources of selected volatile organic compounds (VOCs).

VOC Compound	Common Exposure Sources
Acrolein	Tobacco smoke, combustion of petroleum fuels, industries where acrolein is used, cooking oil, endogenous [11,12].
Acrylamide	Tobacco smoke, eating carbohydrate-rich foods that are cooked at high temperatures, contaminated well-water, working in the production or use of acrylamide and acrylamide containing products (exposure may occur through skin contact) [11,12,13].
Acrylonitrile	Tobacco smoke, industrial sources or hazardous waste sites [11,12,13].
Benzene	Tobacco smoke, automobile service stations, exhaust from motor vehicles, and industrial emissions [11,12,13].
1-Bromopropane	Dry-cleaning, metal-degreasing solvent [11,13].
1,3-Butadiene	Tobacco smoke, vehicle exhaust, waste incineration, or wood fires, drinking contaminated water near production or waste sites [11,12,13].
Carbon disulfide	Tobacco smoke, manufacturing processing [11,12].
Crotonaldehyde	Tobacco smoke, gasoline and diesel engine exhausts, and smoke from wood burning, naturally occur in some foods [11,12].
*N,N*-Dimethylformamide	Tobacco smoke, building materials, glues [11,12].
Ethylbenzene	Tobacco smoke, burning fossil fuels, industries using ethylbenzene, carpet glues, varnishes, and paints [11,12].
Ethylene oxide	Tobacco smoke, occupational exposure, through use in hospital sterilization or use as a pesticide [11,12,13].
Hydrogen cyanide	Tobacco smoke, food, manufacturing processes, endogenous [11,12].
Propylene oxide	Tobacco smoke, occupational exposure, plastics industry [11,12,13].
Styrene	Tobacco smoke, vehicle exhaust, building materials, manufacturing, foods packaged in polystyrene containers [11,12,13].
Toluene	Tobacco smoke, fossil fuels, industrial solvent, paints, paint thinners [11,12]
Tetrachloroethylene	Dry-cleaning, metal degreasing solvent, contaminant detected at superfund sites, surface and groundwater contaminant [13].
Trichroloethylene	Dry-cleaning, industrial solvent [13].
Vinyl chloride	Tobacco smoke, breathing contaminated air from plastics industries, hazardous waste sites, and landfills. Drinking water from contaminated wells [11,12,13].
Xylene	Tobacco smoke, gasoline, paint, varnish, shellac, rust preventatives [11,12,13].

**Table 2:** Descriptive statistics of the National Children’s Study (NCS) Vanguard 2009–2010 urine VOC subsample (*n* = 488).

Variable	Frequency	
	*N*	%
Observed air freshener		
No	357	73.2%
Yes	131	26.8%
Observed incense		
No	458	93.8%
Yes	30	6.2%
Observed candles		
No	237	48.6%
Yes	251	51.4%
Observed other scented products		
No	373	76.4%
Yes	115	23.6%
Reported gas pumping		
No	256	52.5%
Some days	232	47.5%
Every day	0	0.0%
Reported air freshener use		
No	135	27.7%
Some days	223	45.7%
Every day	130	26.6%
Reported aerosol use		
No	217	44.5%
Some days	173	35.4%
Every day	98	20.1%
Reported paint/varnish use		
No	373	76.4%
Some days	115	23.6%
Every day	0	0.0%
Reported paint thinner use		
No	484	99.2%
Some days	4	0.8%
Every day	0	0.1%
Reported turpentine use		
No	484	99.2%
Some days	4	0.8%
Every day	0	0.1%
3-day average reported cooking time		
None	123	25.2%
<1 h	211	43.2%
1 + h	154	31.2%
Smoking exposure		
None	362	74.2%
Some	93	19.0%
Smoker	33	6.8%
Location		
Rural	243	49.8%
Urban	245	50.2%
Visit observations completed		
T1	344	70.5%
T3-First	144	29.5%

**Table 3:** Detection frequencies and concentration distribution for metabolites included in regression models (*n* = 488).

Parent VOC	Metabolite (Short Name)	Metabolite (Full Name)	Detection Frequency (%)	Percentiles (ng/mL)		Maximum (ng/mL)
				50th	75th	
Acrolein	CEMA	*N-*Acetyl*-S-*(2-carboxyethyl)-l-cysteine	99	71.8	124	2260
	3HPMA	*N-*Acetyl*-S-*(3-hydroxypropyl)-l-cysteine	100	240	403	14,400
Acrylamide	GAMA	*N-*Acetyl*-S-*(2-carbamoyl-2-hydroxyethyl)-l-cysteine	49	<9.4 *	15.61	203
	AAMA	*N-*Acetyl*-S-*(2-carbamoylethyl)-l-cysteine	100	33.3	55.23	582
Acrylonitrile	CYMA	*N-*Acetyl*-S-*(2-cyanoethyl)-l-cysteine	83	1.33	2.22	812
Benzene	PMA	*N-*Acetyl*-S-*(phenyl)-l-cysteine	52	0.642	1.11	12.3
	MU	*t,t*-Muconic acid	92	245	391	4090
1-Bromopropane	BPMA	*N-*Acetyl*-S-*(*n*-propyl)-l-cysteine	99	2.61	9.44	4260
1,3-Butadiene	DHBMA	*N-*Acetyl*-S-*(3,4-dihydroxybutyl)-l-cysteine	100	281	431	1730
	MHBMA3	*N-*Acetyl*-S-*(4-hydroxy-2-buten-1-yl)-l-cysteine	94	6.90	12.1	597
Carbon disulfide	TTCA	2-Thioxothiazolidine-4-carboxylic acid	66	5.91	13.3	483
Crotonaldehyde	HPMMA	*N-*Acetyl*-S-*(3-hydroxypropyl-1-methyl)-l-cysteine	100	342	592	17,700
*N,N*-Dimethylformamide	AMCC	*N-*Acetyl*-S-*(*N-*methylcarbamoyl)-l-cysteine	100	66.6	127	2950
Ethylbenzene, styrene	PGA	Phenylglyoxylic acid	93	208	356	2130
Ethylene oxide, vinyl chloride, acrylonitrile	HEMA	*N-*Acetyl*-S-*(2-hydroxyethyl)-l-cysteine	70	0.963	1.72	33.4
Hydrogen cyanide	SCN¯	Thiocyanate	100	832	1435	19,100
Propylene oxide	2HPMA	*N-*Acetyl*-S-*(2-hydroxypropyl)-l-cysteine	98	44.6	86.1	2660
Styrene	MA	Mandelic acid	99	208	302	2190
	PHEMA	*N-*Acetyl*-S-*(1-phenyl-2-hydroxyethyl)-l-cysteine + *N-*Acetyl*-S-*(2-phenyl-2-hydroxyethyl)-l-cysteine	24	<0.7 *	<0.7 *	9.84
Toluene	BMA	*N-*Acetyl*-S-*(benzyl)-l-cysteine	99	5.62	12.1	519
Xylene	2MHA	2-Methylhippuric acid	95	21.2	41.8	3810
	3MHA + 4MHA	3-Methylhippuric acid & 4-methylhippuric acid	100	150	306	17,800

* Below LOD. Parent VOC, refers to the compound that the metabolite originates from.

**Table 4:** Summary of regression results, adjusted parameter estimates (95% CI), and reference groups (Rf) from regression models of metabolites (log based 10).

Parent VOC (Metabolite Short Name)	Intercept	Smoking			Reported Paint/Varnish		Observed Incense		Study Location		Other Considered Covariates
		None	Some	Smoker	No	Yes	Yes	No	Rural	Urban	
Acrolein (CEMA)	1.5 (1.3, 1.7) *	−0.68 (−0.78, −0.58) *	−0.69 (−0.79, −0.59) *	Rf	—	*—*	*—*	Rf	—	—	Reported cooking time
Acrolein (3HPMA)	0.67 (0.47, 0.87) *	−0.43 (−0.53, −0.33) *	−0.42 (−0.52, −0.32) *	Rf	—	*—*	—	Rf	—	—	Reported cooking time
Acrylamide (GAMA)	0.48 (0.28, 0.68) *	−0.42 (−0.52, −0.32) *	−0.44 (−0.54, −0.34) *	Rf	—	*—*	0.09 (−0.008, 0.19)	Rf	—	—	Observed incense
Acrylamide (AAMA)	−0.04 (−0.33, 0.25) *	−0.46 (−0.58, −0.34) *	−0.45 (−0.57, −0.33) *	Rf	—	—	0.11 (−0.0076, 0.23)	Rf	—	—	Observed incense
Acrylonitrile (CYMA)	0.38 (−0.09, 0.85) *	−1.72 (−1.9, −1.6) *	−1.59 (−1.7, −1.5) *	Rf	—	—	—	Rf	−0.02 (−0.079, 0.039)	Rf	
Benzene (MU)	1.2 (0.91, 1.5) *	−0.11 (−0.27, 0.047)	−0.05 (−0.23, 0.13)	Rf	—	—	—	Rf	0.03 (−0.048, 0.11)	Rf	Reported gas pumping
Benzene (PMA)	−1.3 (−1.6, −0.97) *	−0.02 (−0.16, 0.12)	−0.1 (−0.26, 0.057)	Rf	—	—	—	Rf	−0.01 (−0.088, 0.068)	Rf	Reported gas pumping
1,3−Butadiene (DHBMA)	0.87 (0.73, 1) *	−0.11 (−0.17, −0.051) *	−0.15 (−0.21, −0.091) *	Rf	—	—	0.04 (−0.019, 0.099)	Rf	—	—	Observed air freshener, Observed candles, Observed incense, Observed other scented products, Reported air fresheners, Reported cooking time
1,3−Butadiene (MHBMA3)	−0.16 (−0.43, 0.11) *	−0.97 (−1.1, −0.83) *	−1.01 (−1.1, −0.89) *	Rf	—	—	−0.06 (−0.2, 0.077)	Rf	—	—	Observed air freshener, Observed candles, Observed incense, Observed other scented products, Reported air fresheners, Reported cooking time
1−Bromopropane (BPMA)	−0.63 (−1.3, −0.0028) *	−0.15 (−0.44, 0.14) *	−0.33 (−0.66, 0.0032) *	Rf	—	—	0.01 (−0.3, 0.32)	Rf	—	—	Observed incense
Carbon disulfide (TTCA)	−0.79 (−1.2, −0.34) *	−0.12 (−0.32, 0.076)	−0.12 (−0.34, 0.096)	Rf	—	—	—	Rf	—		
Crotonaldehyde (HPMMA)	1.7 (1.5, 1.9) *	−0.69 (−0.77, −0.61) *	−0.71 (−0.81, −0.61) *	Rf	—	—	—	Rf	—	—	
Cyanide (SCN−)	2.7 (2.4, 2.9) *	−0.67 (−0.79, −0.55) *	−0.7 (−0.84, −0.56) *	Rf	—	—	—	Rf	—	—	
Ethylbenzene, styrene (PGA)	0.31 (−0.023, 0.64) *	−0.14 (−0.3, 0.017)	−0.17 (−0.35, 0.0064)	Rf	—	*—*	—	Rf	0.06 (−0.018, 0.14)	Rf	
Ethylene oxide, vinyl chloride, acrylonitrile (HEMA)	−0.58 (−0.85, −0.31) *	−0.6 (−0.72, −0.48) *	−0.56 (−0.7, −0.42) *	Rf	—	—	—	Rf	−0.03 (−0.089, 0.029)	Rf	
*N,N*−Dimethylformamide (AMCC)	0.99 (0.75, 1.2) *	−0.71 (−0.81, −0.61) *	−0.73 (−0.83, −0.63) *	Rf	—	—	—	Rf	—	—	
Propylene oxide (2HPMA)	0.68 (0.35, 1) *	−0.4 (−0.56, −0.24) *	−0.47 (−0.65, −0.29) *	Rf	—	—	—	Rf	—	—	
Styrene (MA)	1 (0.86, 1.2) *	−0.16 (−0.24, −0.082) *	−0.18 (−0.26, −0.1) *	Rf	Rf	0.02 (−0.019, 0.059)	0.03 (−0.048, 0.11)	Rf	—	—	Observed air freshener, Observed candles, Observed incense, Observed other scented products, Reported air fresheners, Reported gas pumping
Styrene (PHEMA)	−1.2 (−1.7, −0.76) *	−0.34 (−0.5, −0.18) *	−0.42 (−0.6, −0.24) *	Rf	Rf	0.09 (−0.008, 0.19)	−0.01 (−0.19, 0.17)	Rf	—	—	Observed air freshener, Observed candles, Observed incense, Observed other scented products, Reported air fresheners, Reported gas pumping
Toluene (BMA)	−1.1 (−1.4, −0.74) *	0.14 (−0.017, 0.3) *	0.08 (−0.096, 0.26)	Rf	Rf	0.02 (−0.058, 0.098)	—	—	−0.12 (−0.2, −0.042) *	Rf	Reported gas pumping
Xylene (2MHA)	0.7 (0.41, 0.99) *	−0.57 (−0.71, −0.43) *	−0.63 (−0.77, −0.49) *	Rf	Rf	0.13 (0.052, 0.21) *	−0.08 (−0.22, 0.057)	Rf	−0.04 (−0.12, 0.038)	Rf	Observed air freshener, Observed candles, Observed incense, Observed other scented products, Reported aerosols, Reported air fresheners, Reported gas pumping
Xylene (3MHA + 4MHA)	1.1 (0.83, 1.4) *	−0.56 (−0.7, −0.42) *	−0.65 (−0.79, −0.51) *	Rf	Rf	0.13 (0.052, 0.21) *	0.08 (−0.057, 0.22)	Rf	0.02 (−0.039, 0.079)	Rf	Observed air freshener, Observed candles, Observed incense, Observed other scented products, Reported aerosols, Reported air fresheners, Reported gas pumping

Notes: Parameter estimates not shown for variables that were not significant in any model. * denotes variable significant at *p* < 0.01. All models included adjustment for visit type and creatinine.

## Abbreviations

The following abbreviations are used in this manuscript:

1DCVMA: *N*-Acetyl-*S*-(1,2-dicholorovinyl)-l-cysteine
2DCVMA: *N*-Acetyl-*S*-(2,2-dicholorovinyl)-l-cysteine
2HPMA: *N*-Acetyl-*S*-(2-hydroxypropyl)-l-cysteine
2MHA: 2-Methylhippuric acid
3HPMA: *N*-Acetyl-*S*-(3-hydroxypropyl)-l-cysteine
3MHA + 4MHA: 3-Methylhippuric acid & 4-methylhippuric acid
AAMA: *N*-Acetyl-*S*-(2-carbamoylethyl)-l-cysteine
AMCC: *N*-Acetyl-*S*-(*N*-methylcarbamoyl)-l-cysteine
BMA: *N*-Acetyl-*S*-(benzyl)-l-cysteine
BPMA: *N*-Acetyl-*S*-(*n*-propyl)-l-cysteine
CEMA: *N*-Acetyl-*S*-(2-carboxyethyl)-l-cysteine
CYMA: *N*-Acetyl-*S*-(2-cyanoethyl)-l-cysteine
DHBMA: *N*-Acetyl-*S*-(3,4-dihydroxybutyl)-l-cysteine
DPMA: *N*-Acetyl-*S*-(2,4-dimethylphenyl)-l-cysteine + *N*-Acetyl-*S*-(2,5-dimethylphenyl)-l-cysteine + *N*-Acetyl-*S*-(3,4-dimethylphenyl)-l-cysteine
GAMA: *N*-Acetyl-*S*-(2-carbamoyl-2-hydroxyethyl)-l-cysteine
HEMA: *N*-Acetyl-*S*-(2-hydroxyethyl)-l-cysteine
HPMMA: *N*-Acetyl-*S*-(3-hydroxypropyl-1-methyl)-l-cysteine
MA: Mandelic acid
MHBMA1: *N*-Acetyl-*S*-(1-hydroxymethyl-2-propenyl)-l-cysteine
MHBMA2: *N*-Acetyl-*S*-(2-hydroxy-3-butenyl)-l-cysteine
MHBMA3: *N*-Acetyl-*S*-(4-hydroxy-2-buten-1-yl)-l-cysteine
MU: *t,t-*Muconic acid
NCS: National children’s study
PGA: Phenylglyoxylic acid
PHEMA: *N*-Acetyl-*S*-(1-phenyl-2-hydroxyethyl)-l-cysteine + *N*-Acetyl-*S*-(2-phenyl-2-hydroxyethyl)-l-cysteine
PMA: *N*-Acetyl-*S*-(phenyl)-l-cysteine
SCN: Thiocyanate
TCVM: *N*-Acetyl-*S*-(trichlorovinyl)-l-cysteine
TTCA: 2-Thioxothiazolidine-4-carboxylic acid
VOCs: Volatile organic compounds
